# Primary intraosseous meningioma with subcutaneous and dural invasion: A case report and literature review

**DOI:** 10.3389/fsurg.2022.995986

**Published:** 2022-10-18

**Authors:** Mingang Zou, Ruijin Yang, Zhiji Tang, Defang Luo, Qiuhua Jiang

**Affiliations:** Department of Neurosurgery, Ganzhou People’s Hospital, Ganzhou, China

**Keywords:** intraosseous meningiomas, skull, osteogenesis, surgical techniques, case report

## Abstract

Primary intraosseous meningiomas (PIOMs) are a rare subset of meningiomas, comprising fewer than 1% of all such tumors. Furthermore, PIOMs presenting as osteogenic lesions that invade both the dura and subcutaneous tissue are extremely rare. Unlike intracranial meningiomas, diagnosing and treating PIOMs are challenges due to their insidious clinical behavior and a lack of clear radiological diagnostic criteria. We report the case of a 60-year-old female with headache and a slightly outward protrusion of the parietal region of the skull. CT showed an osteogenic lesion in the right parietal bone. MR imaging indicated mild to moderate homogeneous enhancement with an intense dural reaction. The suggested clinical diagnosis was lymphoma, so we performed a skull biopsy, which revealed an intraosseous benign meningioma. A precise resection strategy was planned with a neuronavigation system accompanied by a one-step customized titanium mesh cranioplasty. The lesion was completely removed, and pathological analysis confirmed a meningothelial meningioma (WHO Grade I) of intraosseous layer origin invading the dura mater and subcutaneous tissue. This case highlights the need for an initial biopsy when the lesion is difficult to diagnose on imaging. Complete resection should be attempted to minimize the risk of recurrence.

## Introduction

Primary epidural meningiomas (PEMs), once defined as entities that develop in the extradural compartments, account for less than 2% of all meningiomas (1). Primary intraosseous meningiomas (PIOMs), which arise from bone and especially the skull, are a subtype of epidural meningioma that has only been reported in a few cases (2). It is extremely rare for a PIOM to exhibit radiographic features of both osteogenesis and dural invasion (3, 4), and such PIOMs are often misdiagnosed as primary bone tumors and en plaque meningiomas. Here, we report the surgical case of a primary intraosseous osteoblastic meningioma with subcutaneous and dural infiltration and review the literature.

## Case presentation

**Figure 1:**
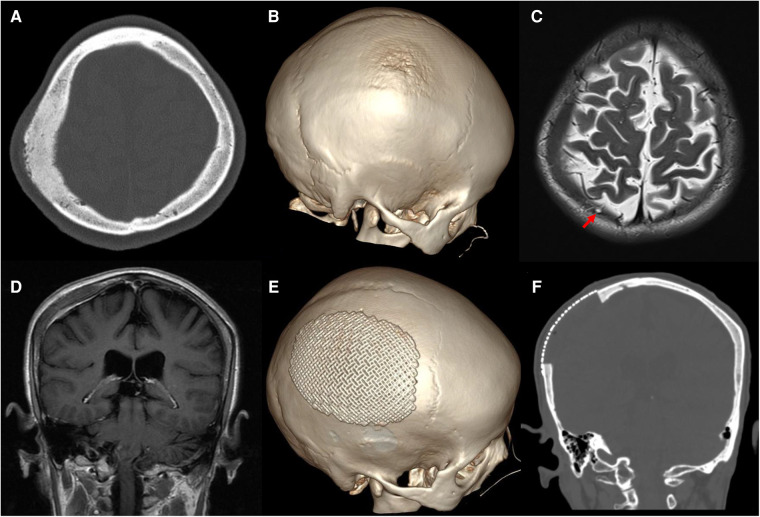
Preoperative images: (**A**) axial thin-cut skull CT shows an osteogenic lesion in the right parietal bone. (**B**) 3D-CT shows crater-like changes in the outer layer of the skull. Axial T2 imaging revealed a hypointense intraosseous lesion and arachnoid granules protruding into the diseased bone [(**C**), red arrow]. (**D**) Coronal gadolinium-enhanced MR imaging exhibits mild to moderate homogeneous enhancement with an intense dural reaction. Postoperative CT shows total resection of the tumorized calvaria lesion with good coverage by a titanium mesh (**E,F**).

A 60-year-old woman was admitted to our neurosurgery department for headache and a 6-month history of a skull mass in the right parietal region. No trauma had previously occurred in that region. Neurological examination and laboratory data showed no significant abnormalities. Thin-cut skull computed tomography (CT) showed a large parietal osteogenic calvarial lesion measuring 7.1 × 5.8 × 2.2 cm (Figure 1A). The lesion involved the full layer of the parietal bone with crater-like changes in the outer layer of the skull (Figure 1B). Further T2-weighted magnetic resonance (MR) imaging revealed a hypointense intraosseous lesion (Figure 1C). Gadolinium-enhanced MR imaging presented mild to moderate homogeneous enhancement with an intense dural reaction (Figure 1D).

Considering the possibility of lymphoma according to the radiologist’s assessment, we performed a skull biopsy in the most prominent area of the lesion. An arc incision was adopted in case of possible subsequent surgery. The result confirmed an intraosseous ectopic meningioma.

**Figure 2:**
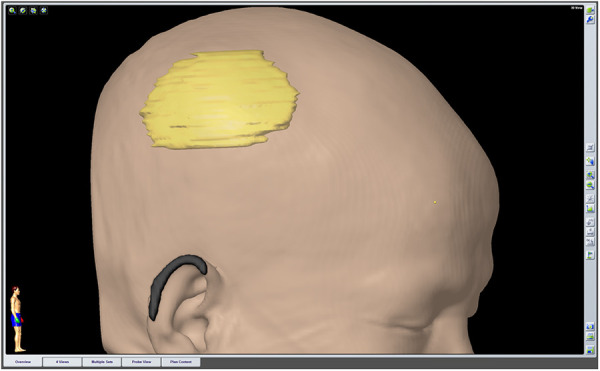
The extent of the preoperatively planned resection was outlined by the neuronavigation system before surgery.

As there were no neurological deficits, 2 weeks after the biopsy, precise resection of the lesion and a cranioplasty were performed. Before the operation, the approximate extent of the resection was determined by the neuronavigation system (Figure 2). The information obtained during 3D thin-slice CT reconstruction was shared with the manufacturer to customize an individualized titanium mesh to provide good function and cosmetic effect. After the skin flap was peeled during surgery, the skull lesion did not show strong adhesion to the scalp, but it had partially infiltrated the subcutaneous tissue. Roughness of the outer plate and normal levels of cancellous bone loss in the diploic space were observed. The dura was thickened with a perforation in the center, and a layer of readily dissected gelatinous tissue appeared on the surface of the parietal lobe without brain tissue invasion. Then, the lesion involving the scalp, skull, and dura was completely resected with the aid of neuronavigation. The defect of the dura mater was repaired with normal autologous fascia, and dural tenting sutures were performed to prevent epidural effusion. Then, we completed the cranioplasty.

**Figure 3:**
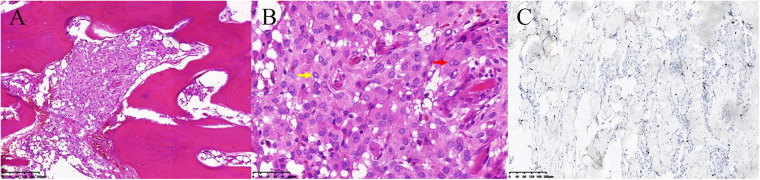
Pathology of the primary intraosseous meningioma. (**A**) The intertrabecular spaces had been infiltrated by meningioma cells (hematoxylin / eosin staining, 100×). (**B**) The cells had differentiated from meningothelial cells as syncytia (red arrow), with some intranuclear inclusions (yellow arrow) and exhibited a low Ki-67 proliferation rate (**C**).

Postoperative CT showed total resection of the tumorous calvaria lesion with complete restoration of the symmetry of the skull (Figures 1E,F). Microscopic examination of H/E-stained sections revealed that the intertrabecular spaces had been infiltrated by tumor cells with eosinophilic cytoplasm and round nuclei, which extended into the subcutaneous space and dura (Figure 3A). The cells were differentiated from meningothelial cells as syncytia, with some intranuclear inclusions (Figure 3B). According to the overall features, the lesion was identified as a meningothelial-type meningioma (WHO Grade I). The Ki-67 labeling index was 5%, indicating a low proliferative potential (Figure 3C). The recovery course was uneventful. After follow-up for 3 months, no recurrence or other complaints were noted.

## Discussion

Most PEMs are considered to be rare tumors involving bone and subcutaneous tissue, of which approximately two-thirds are PIOMs. The incidence of these latter meningiomas peaks at 60 years of age, with a significant female predominance (85%) (5). The frontoparietal and orbital regions are the most common sites of PIOMs (6). Patients usually exhibit no neurological symptoms but only a local, slow-growing bulge. Ophthalmoplegia or visual field defects may be present when the tumor is located in the skull base (7).

The presence of certain tumors invading the dura during surgery raises the question of whether they should be classified as PEMs. Bassiouni et al. (3) reported that 14 of 16 PEM patients (88%) who underwent surgery exhibited dural involvement by pathology. In another report, there appeared to be no evidence of dural invasion by macroscopic examination during surgery, but subsequent pathology confirmed tumor infiltration of both layers of the dura (8). Recently, Ahmed et al. (9) proposed a new classification of PIOMs, which included type IV (mixed variety), defined as tumors extending from the dura to the extracalvular space. Therefore, dural resection and duraplasty were performed in our case. Identifying the location of the main area of the tumor within the skull can be performed to determine its origin and specify whether it is a PIOM or PEM, regardless of the presence of dural invasion. According to a review of a number of studies (4, 10, 11), PIOMs tend to form a broader base in the skull than in the dura, whereas tumors of meningeal origin, such as meningiomas, have a wider base in the dura than in the skull. Therefore, we considered our case to be a PIOM involving the dura and soft tissue rather than an intracranial meningioma involving the bone and soft tissue.

To date, several hypotheses have been proposed regarding the origin of PIOMs. They may be derived from the abnormal differentiation and migration of mesenchymal stem cells (12). Additionally, blood vessels and nerves penetrating the skull may carry arachnoid cap cells to different sites and subsequently proliferate (7). Other theories suggest that arachnoid cap cells can be captured in the posttraumatic fracture line or cranial suture during cranium growth (13). In our case, arachnoid granules protruding into the diseased parietal bone were observed on MR imaging, which may explain the origin of some intraosseous meningiomas.

In one study, the imaging characteristics of PIOMs included hyperostosis in 56% of the meningiomas, osteolysis in 33%, and mixed features in 11% (14). These features may be due to neoplastic changes occurring within the skull that stimulate osteogenic or osteoclast activity to produce various stages of bone response and bone resorption (5). These features also present a challenge for the radiological diagnosis of PIOMs. Osteomas, primary cranial lymphoma, fibrous dysplasia, eosinophilic granulomas, and metastatic tumors are often part of the differential diagnosis (4, 14, 15). In our case, we observed osteoblastic hyperplasia in the CT bone window. MRI is valuable for identifying extraosseous involvement. We observed dural enhancement in our patient, which is distinct from the dural tail sign of intracranial meningiomas. The preoperative diagnosis was mistaken for primary cranial lymphoma because of its similar radiological appearance to PIOM, but the treatment of the former mainly includes radiotherapy and chemotherapy (16). The final diagnosis of PIOM was confirmed by pathology, suggesting the importance of histopathological biopsy. Biopsy is appropriate when the skull lesion is difficult to diagnose on imaging. This biopsy can be performed under local anesthesia with minimal trauma, but the incision line design needs to take into account possible subsequent surgical resection. Intraosseous meningiomas are mostly benign. However, they often extend both intraosseously and extraosseously when presenting as malignant growths (17). In general, the osteoblastic type has a lower incidence of atypical or malignant features than the osteolytic type (2). Even if histology is benign, PIOMs may appear to be highly aggressive. In this case, the osteoblasts of the skull invaded the subcutaneous tissue and dura and spread to the surface of the parietal lobe as a layer of gelatinous tissue without breaking through the pia mater, which is extremely rare. According to the latest criteria, atypical meningioma (WHO Grade II) is considered if the tumor cells penetrate the pia mater and cause brain invasion (18). Therefore, in this case, there is a tendency for the lesion to deteriorate further.

Wide surgical excision is the primary treatment of choice for PIOMs (9). The literature has reported a 22% probability of the recurrence of benign PIOMs, and lesions located at the skull base have a higher recurrence rate than those located at the convex surface, which may be related to incomplete resection (1, 14). Therefore, complete resection should be attempted, including for any affected tissue that may contain tumor cells, which may result in cure. Nowak-Choi K et al. (19) found that Ki-67 labeling index values of WHO grade I meningiomas that are above 5% indicated high proliferative activity and high risk for recurrence. The patient in our case may have been at risk for recurrence, as the index was 5%. We used the neuronavigation system to outline the preresection line before surgery and relied on the guidance of a sterile navigation pen to precisely and maximally remove the lesioned skull. The extent of resection, which was at least 10 mm beyond the periphery of the lesion, was consistent with the safe distance reported in the literature (20). The involved dura and subcutaneous tissue were also resected. Additionally, computer-aided reconstruction technology was used to customize the titanium mesh individually for the planned resection area to complete the cranioplasty in one stage, achieving a good aesthetic effect. Titanium mesh was selected for our cases due to its low cost, aesthetic effect and low infection rate, despite its poor thermostability and deformation under trauma. The dural defect was reconstructed by watertight suturing of the temporal muscle fascia rather than artificial meningeal application for the following reasons. First, the former can reduce the probability of chronic inflammatory reactions and has better histocompatibility. Second, the use of artificial dura mater is more prone to epidural effusion. In addition, the high cost of artificial dura mater was also considered. Of course, our case has the limitation of short postoperative follow-up time.

## Conclusion

We encountered a rare case of osteoblastic intraosseous meningioma that originated from the cranial diploic layer and invaded the dura mater and subcutaneous tissue. PIOMs may appear to be highly aggressive even if the histology is benign. To prevent recurrence and deterioration, surgery should be performed to achieve complete resection of the lesion. The clinical features and surgical techniques described in this report may provide a good reference for the diagnosis and treatment of similar diseases in the future.

## Data Availability

The original contributions presented in the study are included in the article/Supplementary Material, further inquiries can be directed to the corresponding author/s.
